# Love after lockup: examining the role of marriage, social status, and financial stress among formerly incarcerated individuals

**DOI:** 10.1186/s40352-024-00264-x

**Published:** 2024-02-24

**Authors:** Jemar R. Bather, Anna-Michelle Marie McSorley, Brennan Rhodes-Bratton, Adolfo G. Cuevas, Saba Rouhani, Ridwan T. Nafiu, Adrian Harris, Melody S. Goodman

**Affiliations:** 1 Center for Anti-Racism, Social Justice & Public Health, New York University School of Global Public Health, 708 Broadway, 9th Floor, New York, NY 10003 USA; 2 Department of Biostatistics, New York University School of Global Public Health, New York, NY 10003 USA; 3 Department of Social and Behavioral Sciences, New York University School of Global Public Health, New York, NY 10003 USA; 4 Department of Epidemiology, New York University School of Global Public Health, New York, NY 10003 USA; 5 Center for Drug Use and HIV/HCV Research, New York University School of Global Public Health, New York, NY 10003 USA

**Keywords:** Formerly incarcerated individuals, Criminal record, Marital status, Social status, Incarceration, Financial stress, Reintegration, Prison, Psychological distress, Financial strain

## Abstract

**Background:**

Upon reintegration into society, formerly incarcerated individuals (FIIs) experience chronic financial stress due to prolonged unemployment, strained social relationships, and financial obligations. This study examined whether marriage and perceived social status can mitigate financial stress, which is deleterious to the well-being of FIIs. We also assessed whether sociodemographic factors influenced financial stress across marital status. We used cross-sectional data from 588 FIIs, collected in the 2023 Survey of Racism and Public Health. The financial stress outcome (Cronbach’s $\mathrm{\alpha }$ = 0.86) comprised of five constructs: psychological distress, financial anxiety, job insecurity, life satisfaction, and financial well-being. Independent variables included marital and social status, age, race/ethnicity, gender identity, educational attainment, employment status, and number of dependents. Multivariable models tested whether financial stress levels differed by marital and perceived social status (individual and interaction effects). Stratified multivariable models assessed whether social status and sociodemographic associations varied by marital status.

**Results:**

We found that being married/living with a partner (M/LWP, b = -5.2) or having higher social status (b = -2.4) were protective against financial stress. Additionally, the social status effect was more protective among divorced, separated, or widowed participants (b = -2.5) compared to never married (NM, b = -2.2) and M/LWP (b = -1.7) participants. Lower financial stress correlated with Black race and older age, with the age effect being more pronounced among M/LWP participants (b = -9.7) compared to NM participants (b = -7.3). Higher financial stress was associated with woman gender identity (overall sample b = 2.9, NM sample b = 5.1), higher education (M/LWP sample b = 4.4), and having two or more dependents (overall sample b = 2.3, M/LWP sample b = 3.4).

**Conclusions:**

We provide novel insights into the interrelationship between marriage, perceived social status, and financial stress among FIIs. Our findings indicate the need for policies and programs which may target the family unit, and not only the individual, to help alleviate the financial burden of FIIs. Finally, programs that offer legal aid to assist in expungement or sealing of criminal records or those offering opportunities for community volunteer work in exchange for vouchers specific to legal debt among FIIs could serve to reduce financial stress and improve social standing.

## Introduction

In 2019, approximately 1,700 people were released from prison daily in the United States (Carson, 2020). Upon reintegration into society, formerly incarcerated individuals (FIIs) face a multitude of hardships, including significant legal financial obligations (Ginapp et al., 2023; Harper et al., 2021; Harris et al., 2010; Montes et al., 2021). According to one study, the average debt owed to the courts by FIIs exceeded $13,000 (deVuono-powell et al., 2015). To manage these payments and other living expenses, FIIs often depend on social support systems such as family members, friends, or spouses for financial assistance (Denney et al., 2014; Visher et al., 2004, 2008; Western et al., 2015). However, these relationships can strain over time as FIIs experience extended periods of unemployment (Lindsay, 2022; Montes et al., 2021; Pager, 2003, 2007).

Analyses of the National Longitudinal Survey of Youth estimate that nearly half of unemployed men in the United States have a criminal history (Bushway et al., 2022). Research indicates that FIIs encounter challenges securing employment due to shorter work histories, lower educational attainment, and fewer credentials than those without a criminal background (Wakefield & Uggen, 2010). Findings from a randomized experiment showed that managers preferred hiring individuals without a criminal history, despite being equally qualified as an individual with a criminal history (Santos et al., 2023). Insufficient funding often hinders the effectiveness of interventions designed to increase employment among the unemployed (Edelman & Holzer, 2013). Even when FIIs secure employment, the position may offer inadequate compensation, not align with their skillset, or involve physically demanding tasks (Lindsay, 2022; Pager, 2003; Western, 2002; Western & Beckett, 1999). These employment challenges combined with other barriers such as loss of eligibility to apply for certain jobs, licences, student loans, and public benefits can result in substantive financial hardship among FIIs (Chin, 2012; Lerman & Weaver, 2014; Manza & Uggen, 2006; Mauer & Chesney-Lind, 2002; Rubinstein & Mukamal, 2001; Stafford, 2006).

Spouses often provide emotional and financial support to combat these hardships as FIIs reintegrate into society (Bannon et al., 2010; McKay et al., 2016; Mowen & Visher, 2016). However, literature suggests that these marriages are at an increased risk for divorce due to various factors (Apel et al., 2010; Geller, 2013; Lopoo & Western, 2005; Massoglia et al., 2011; Western, 2006; Western et al., 2004; Widdowson et al., 2020). Spouses may become weary from constantly supporting their partner through emotional and psychological challenges. FIIs often internalize stress, trauma, anxiety, and depression endured during their time in prison from negative interactions with correctional officers and fellow inmates (Binswanger et al., 2007; Visher et al., 2004). Also, the financial burden of incarceration can stress the marital relationship, leading to conflicts about money (Visher et al., 2004). For instance, marital problems may be more likely to arise in larger households with high unmet financial demands or under conditions created by prolonged unemployment, which compound chronic stress (Kraft, 2001; Twenge et al., 2003). This is supported by longitudinal analyses providing evidence that FIIs tend to be economically worse off post-release than before incarceration (Lösel et al., 2012; Markson et al., 2015; Souza et al., 2015). These analyses also highlight the demanding role of the spouse or partner in supporting a FII (Lösel et al., 2012; Markson et al., 2015; Souza et al., 2015). Souza et al. (2015) found that ex-partners of FIIs reported more post-release relationship problems than they initially anticipated during pre-release interviews. Spouses and partners may also witness their formerly incarcerated partner exhibiting cruel discipline towards their children (Markson et al., 2015). Another reason that these relationships may be at increased risk for separation or dissolution is that the spouse may be stigmatized and receive less social support from family members and friends as result of being married to a FII (Braman, 2004; Keene et al., 2018; Turney et al., 2012). The spouse may also constantly worry about their partner reoffending, leading to reincarceration (Goffman, 2009). These tensions and conflicts within the marriage can culminate in divorce, potentially leaving FIIs without financial support from their ex-spouse. Additionally, FII may perceive themselves as having lower societal status after the marriage is dissolved (Porter, 2014).

Perceived social ranking, or subjective social status (Adler et al., 2000), is important to examine among FIIs, particularly within the context of marriage and financial stress. It may serve as a protective factor among FIIs, as having a spouse and higher perceived social status can provide access to financial resources and professional networks (Conger et al., 2010). Research indicates that higher perceived social status is linked to better self-rated physical health (Ostrove et al., 2000). There is also evidence that subjective social status measures (e.g., MacArthur Scale) predict health and well-being outcomes better than traditional socioeconomic indicators, such as income and education (Garza et al., 2017; Singh-Manoux et al., 2005; Tan et al., 2020). In addition, subjective social status measures tap into constructs not captured by income and education variables (Galvan et al., 2022). Hence, analyzing perceived social status levels across different marital status groups may help us elucidate disparities in financial stress among FIIs and identify intervention opportunities. This is critical since medical issues among FIIs often go untreated due to financial hardship (Begun et al., 2016). Therefore, we analyzed a subsample of 588 FIIs from the 2023 Survey of Racism and Public Health to test associations between marriage, social status, and financial stress. We also assessed whether sociodemographic factors influenced financial stress across marital status groups.

## Methods

### Study population

We hired Qualtrics Research Services (QRS) to recruit participants, collect online survey data, and provide incentives to participants. The web-based cross-sectional survey collected sociodemographic information, self-reported experiences with discrimination, social status, financial and food insecurity, voting practices, policing experiences, and health. Our team provided QRS with the survey URL and back-end access to an institutional account to host and manage the data collection. Our research team did not recruit or interact with participants. QRS recruited qualified respondents from Qualtrics’ panel sources, based on the inclusion and exclusion criteria and sampling quota. Criteria for selecting the participants were as follows: 18 years or older, English-speaking/reading, and living in Connecticut, Delaware, District of Columbia, Maine, Maryland, Massachusetts, New Hampshire, New Jersey, New York, Pennsylvania, Puerto Rico, Rhode Island, Vermont, or Virginia (areas within Health and Human Services Regions 1, 2, or 3). The samling quotas provided to QRS were demographic variables that oversampled racial/ethnic minoritized groups. The following demographic quotas were requested from QRS: age (30% aged 18–34, 32% aged 35–54, and 38% aged 55 or older) and racial/ethnic (50% White, 20% Black, 20% Latinx, and 10% Other) groups. The survey went live in March 2023 and closed in April 2023 once the target demographic quotas were met. Profiling data for online survey participants were collected using larger internal surveys that were interspersed between client surveys according to QRS’ standard panel management practices. Panelists have joined the QRS panel through hundreds of different recruitment sources, including direct sign-up, co-registration offers on partners’ websites, targeted emails sent by online partners to their audiences, graphical and text banner placement on partners’ websites, trade show presentations, targeted postal mail invitations, TV advertisements, member referrals, and telephone recruitment of targeted populations. The survey consisted of 84 questions and the average time to survey completion was 15 min.

**Fig. 1:**
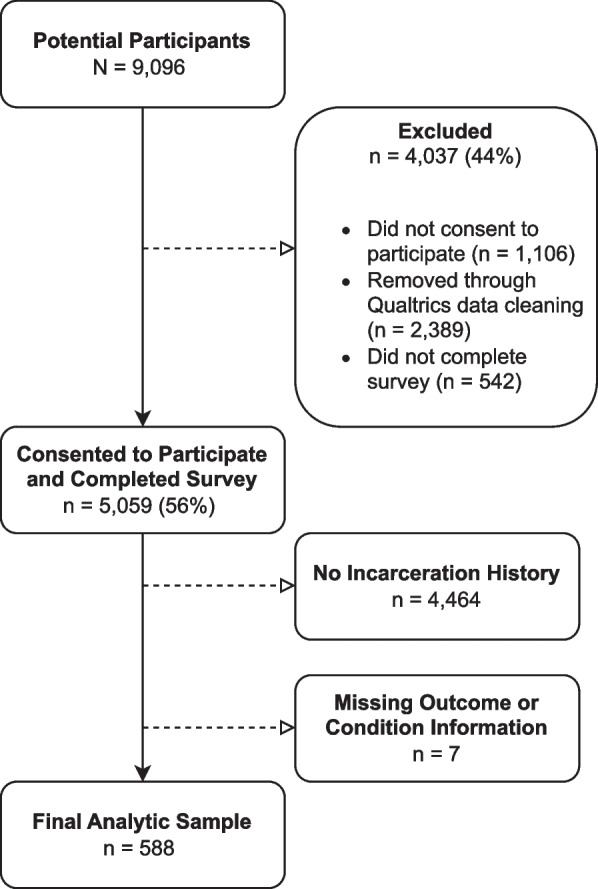
Recruitment flow diagram, Survey of Racism and Public Health, 2023

Over 9,000 individuals (*N* = 9,096) were recruited to complete the 2023 Survey of Racism and Public Health (Fig. 1). Of these, 44% did not consent to participate (*n* = 1,106), did not complete the survey (*n* = 542), or were removed through QRS data cleaning (*n* = 2,389). Reasons for removal through data cleaning included: non-sensical answers, duplicates, bots, contradictory responses, infeasible response values for weight or height, and invalid IP address. The remaining 56% (*n* = 5,059) consented to participate and completed the survey. Among these 5,059 study participants, 4,464 were excluded because of no incarceration history, and seven individuals were excluded because of missing outcome or condition information (outcome: *n* = 6, marital status: *n* = 1). The final analytic sample was selected based on participants who self-reported “yes” to “Have you ever been incarcerated?” This resulted in a sample of 588 FIIs. All study participants were compensated with cash, loyalty rewards, or gift cards by a QRS third-party vendor.

### Financial stress

We evaluated financial stress using an adapted version of the APR Financial Stress Scale (Heo et al., 2020), which consists of five constructs: psychological distress, financial anxiety, job insecurity, life satisfaction, and financial well-being (Table 1). Psychological distress was measured with the six-item Kessler Psychological Distress Scale (K6) (Kessler et al., 2002), assessing feelings of sadness, nervousness, restlessness, hopelessness, effortfulness, and worthlessness over the past 30 days. Participants rated each item on a 5-point Likert scale ranging from *(1) none of the time* to *(5) all of the time* (Cronbach’s $\mathrm{\alpha }$ = 0.91). The remaining constructs (financial anxiety, job insecurity, life satisfaction, and financial well-being) used a 5-point Likert scale from *(1) strongly disagree* to *(5) strongly agree.* Financial anxiety was measured using two items from the Financial Anxiety Scale (Archuleta et al., 2013): “I feel anxious about my financial situation” and “I worry about my financial situation” (Cronbach’s $\mathrm{\alpha }$ = 0.87). Job insecurity was assessed using one item developed by Hellgren et al. (1999): “I am worried about having to leave my job before I would like to”. We measured life satisfaction using two items from the Satisfaction With Life Scale (Diener et al., 1985): “In most ways my life is close to my ideal” and “If I could live my life over, I would change almost nothing” (Cronbach’s $\mathrm{\alpha }$ = 0.71). Both life satisfaction items were reverse coded. We assessed financial well-being using four items derived from the Consumer Financial Protection Bureau Well-Being Scale (Consumer Financial Protection Bureau, 2015): “I could handle a major unexpected expense” (reverse coded); “Because of my money situation, I feel like I will never have the things I want in life”; “I can enjoy life because of the way I am managing my money” (reverse coded); and “Giving a gift for a wedding, birthday or other occasion would put a strain on my finances for the month” (Cronbach’s $\mathrm{\alpha }$ = 0.69). Scores for each of the five constructs were summed to create a financial stress index (range: 18–72). Higher scores indicated greater financial stress. The overall financial stress index comprised of all five constructs demonstrated high reliability (Cronbach’s $\mathrm{\alpha }$ = 0.86) (Tavakol & Dennick, 2011).

**Table 1:** Components of the financial stress index, Survey of Racism and Public Health, 2023

Dimension	Validated Scale	# Items	Assessment	Cronbach’s $\boldsymbol{\alpha }$
Financial Stress	APR Financial Stress Scale (Heo et al., 2020)	15	Affective reactions, relational behavior, and physiological responses to financial stress	0.86
*Subscales*				
Psychological distress	Kessler Psychological Distress Scale (Kessler et al., 2002)	6	Risk for severe mental disorders	0.91
Financial anxiety	Financial Anxiety Scale (Archuleta et al., 2013)	2	Heightened financial distress levels	0.87
Job insecurity	Measure of Quantitative Insecurity (Hellgren et al., 1999)	1	Perceived threat to continued employment	–
Life satisfaction	Satisfaction With Life Scale (Diener et al., 1985)	2	Overall judgment of one’s life	0.71
Financial well-being	Consumer Financial Protection Bureau Well-Being Scale (Consumer Financial Protection Bureau, 2015)	4	The degree to which one’s financial condition and capability have offered them a sense of security and freedom	0.69

### Marital and social status

Marital status was categorized as married/living with a partner, divorced/separated/widowed, or never married. We assessed perceived social status using the MacArthur Scale of Subjective Social Status – Adult Version (Adler et al., 2000). Participants ranked themselves from *1 (lowest)* to *10 (highest)* on the following question: “Think of this ladder as representing where people stand in our society. At the top of the ladder are the people who are the best off, those who have the most money, the most education, and the best jobs. At the bottom are the people who are the worst off, those who have the least money, the least education, and the worst jobs or no job. Where would you place yourself on this ladder? Please select the number that best represents where you would be on this ladder.” Higher scores indicated higher perceived social status.

### Sociodemographic characteristics

We recoded the following covariates for this analysis: age (quartiles: 18–36, 37–44, 45–57, 58–90), race/ethnicity (White, Black, Latinx, Multiracial/Other), gender identity (man, woman), educational attainment (≤ high school, some college, ≥ college degree), employment status (full/part-time, other), and number of dependents (0, 1, 2 +). Participants who identified as non-Hispanic American Indian, Native American, Arab, Middle Eastern, North African, Asian American, or Pacific Islander were categorized as “other” race/ethnicity. Those on temporary leave, unemployed, not working by choice, independent contractors, or business owners were categorized as the “other” employment status group.

### Analytic strategy

Descriptive statistics were calculated for all variables. Kruskal–Wallis rank sum tests and Pearson’s Chi-squared tests were conducted to identify differences in characteristics by marital status. We used multivariable linear regression models to estimate the individual and combined effects of marital and social status (Hidalgo & Goodman, 2013). We also stratified these models to assess whether effects of social status and sociodemographics differed by marital status. We presented beta estimates with 95% confidence intervals. We used R (R Core Team, 2023) to perform all statistical analyses and set statistical significance at a *p*-value of < 0.05.

## Results

### Study population characteristics

The 588 respondents had a mean age of 46 (SD = 14) and were primarily White (44%) or Black (28%) and idenitified as a man (69%) (Table 2). Of the 588 respondents, 47% were married/living with a partner, 33% were never married, and 20% were divorced/separated/widowed. Many of the respondents had some college education (61%), worked full/part-time (57%), and had at least one dependent (53%). The average financial stress score was 46 (SD = 11), and the average social status ranking was 5 (SD = 2).

**Table 2:** Survey of Racism and Public Health study participant characteristics, March 10 to April 12, 2023

*Characteristic*		**Overall**	**Never married**	**Divorced/Separated/Widowed**	**Married/Living with partner**	
	*N*	*N* = *588*	*N* = *193*	*N* = *119*	*N* = *276*	*P-value*^*1*^
**Financial Stress**	588					0.20
Mean (SD)		46.0 (11.0)	47.2 (10.6)	45.5 (10.7)	45.3 (11.3)	
**Social Status**	588					< 0.001
Mean (SD)		5.0 (2.2)	4.5 (2.0)	4.7 (2.1)	5.4 (2.3)	
**Age**	588					< 0.001
Mean (SD)		46.1 (13.9)	41.5 (12.3)	53.4 (12.8)	46.2 (14.1)	
**Age quartiles, n (%)**	588					< 0.001
18–36		158 (27%)	70 (36%)	13 (11%)	75 (27%)	
37–44		136 (23%)	51 (26%)	18 (15%)	67 (24%)	
45–57		155 (26%)	47 (24%)	39 (33%)	69 (25%)	
58–90		139 (24%)	25 (13%)	49 (41%)	65 (24%)	
**Race/ethnicity, n (%)**	586					< 0.001
White		255 (44%)	63 (33%)	60 (50%)	132 (48%)	
Black		167 (28%)	75 (39%)	31 (26%)	61 (22%)	
Latinx		105 (18%)	29 (15%)	19 (16%)	57 (21%)	
Multiracial/Other		59 (10%)	25 (13%)	9 (8%)	25 (9%)	
**Gender identity, n (%)**	584					0.30
Man		404 (69%)	125 (65%)	83 (70%)	196 (72%)	
Woman		180 (31%)	67 (35%)	36 (30%)	77 (28%)	
**Education, n (%)**	583					< 0.001
$\le$ High School		225 (39%)	92 (48%)	45 (38%)	88 (32%)	
Some College		234 (40%)	76 (40%)	50 (43%)	108 (39%)	
$\ge$ College Degree		124 (21%)	24 (13%)	22 (19%)	78 (28%)	
**Employment, n (%)**	586					< 0.001
Other		254 (43%)	80 (41%)	71 (60%)	103 (37%)	
Full/part-time		332 (57%)	113 (59%)	47 (40%)	172 (63%)	
**Dependents, n (%)**	588					< 0.001
0		275 (47%)	117 (61%)	75 (63%)	83 (30%)	
1		141 (24%)	40 (21%)	25 (21%)	76 (28%)	
2 +		172 (29%)	36 (19%)	19 (16%)	117 (42%)	

^1^Kruskal-Wallis rank sum test; Pearson’s Chi-squared test

We identified statistically significant differences in several characteristics across marital status groups. Compared to those who were divorced/separated/widowed (mean age = 53.4 years) or married/living with a partner (mean age = 46.2 years), those who never married tended to be younger (mean age = 41.5 years, *P* < 0.001). Those who were never married (mean = 4.5) also ranked themselves lower on the social status ladder relative to those who were divorced/separated/widowed (mean = 4.7) or married/living with a partner (mean = 5.4, *P* < 0.001). Never married participants (48% with HS diploma) had lower education than those who were divorced/separated/widowed (38%) and married/living with a partner (32%; *P* < 0.001). A greater proportion of those who were divorced/separated/widowed (50%) identified as White (married/living with a partner = 48%, never married = 33%, *P* < 0.001). A greater proportion of those who were married/living with a partner worked full or part time (63%), relative to those who were divorced/separated/widowed (40%) or never married (59%; *P* < 0.001). Participants who were married/living with a partner (42% with 2 + dependents) tended to have more children (never married = 19%, divorced/separated/widowed = 16%, *P* < 0.001).

### Associations with financial stress

**Fig. 2:**
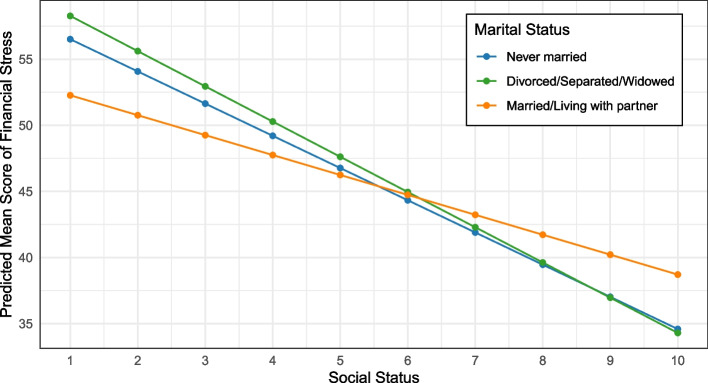
Predicted mean scores of financial stress among formerly incarcerated individuals by marital and social status, adjusting for age, race/ethnicity, gender identity, education, employment status, and number of dependents, Survey of Racism and Public Health, 2023

Table 3 summarizes the adjusted associations between financial stress, marital status, and social status in the overall sample. We observed statistically significant individual and interaction effects of marital and social status. Among the overall sample, lower financial stress correlated with being married/living with a partner (b = -5.2, 95% CI: -9.8, -0.6) and higher perceived social status (b = -2.4, 95% CI: -3.2, -1.7). The significant interaction effect (b = 0.9, 95% CI: 0.1, 1.8) suggests that the association between perceived social status and financial stress varied by marital status. Each increase on the perceived social status ladder translated to a steeper slope (lower financial stress, Fig. 2) among divorced/separated/widowed participants (b = -2.5, 95% CI: -3.4, -1.6) compared to never married (b = -2.2, 95% CI: -2.9, -1.5) and those married/living with a partner (b = -1.7, 95% CI: -2.3, -1.1; Table 4).

**Table 3:** Adjusted associations between financial stress, marital status, and social status among the overall sample of formerly incarcerated individuals, Survey of Racism and Public Health, 2023

Characteristic	Beta	95% CI	*P*-value
**Marital Status**			
Never married	—	—	
Divorced/Separated/Widowed	2.0	-3.7, 7.7	0.50
Married/Living with partner	-5.2	-9.8, -0.6	0.028
**Social Status**	-2.4	-3.2, -1.7	< 0.001
**Age**			
18–36	—	—	
37–44	-3.5	-5.7, -1.2	0.003
45–57	-4.3	-6.6, -2.0	< 0.001
58–90	-7.7	-10.0, -5.1	< 0.001
**Race/ethnicity**			
White	—	—	
Black	-2.9	-5.0, -0.8	0.006
Latinx	-0.4	-2.8, 2.0	0.74
Multiracial/Other	0.2	-2.6, 3.1	0.88
**Gender identity**			
Man	—	—	
Woman	2.9	1.1, 4.7	0.002
**Education**			
$\le$ High School	—	—	
Some College	0.7	-1.1, 2.5	0.46
$\ge$ College Degree	2.2	-0.2, 4.5	0.07
**Employment**			
Other	—	—	
Full/part-time	-0.1	-2.0, 1.6	0.87
**Dependents**			
0	—	—	
1	0.6	-1.5, 2.8	0.59
2 +	2.3	0.1, 4.4	0.041
**Marital Status x Social Status**			
Divorced/Separated/Widowed x Social Status	-0.2	-1.3, 0.9	0.69
Married/Living with partner x Social Status	0.9	0.1, 1.8	0.036
No. Obs	575		

*CI* Confidence Interval

**Table 4:** Adjusted associations between financial stress and social status stratified by marital status among formerly incarcerated individuals, Survey of Racism and Public Health, 2023

*Characteristic*	**Never married**			**Divorced/Separated/Widowed**				**Married/Living with partner**	
	*Beta*	*95% CI*	*P-value*	*Beta*	*95% CI*	*P-value*	*Beta*	*95% CI*	*P-value*
**Social Status**	-2.2	-2.9, -1.5	< 0.001	-2.5	-3.4, -1.6	< 0.001	-1.7	-2.3, -1.1	< 0.001
**Age**									
18–36	—	—		—	—		—	—	
37–44	-3.1	-6.6, 0.4	0.08	2.8	-4.3, 9.9	0.44	-4.9	-8.4, -1.6	0.005
45–57	-2.7	-6.5, 1.1	0.16	1.0	-5.3, 7.3	0.75	-6.9	-10.4, -3.4	0.001
58–90	-7.3	-12.1, -2.4	0.003	-1.9	-8.6, 4.7	0.56	-9.7	-13.8, -5.7	< 0.001
**Race/ethnicity**									
White	—	—		—	—		—	—	
Black	-3.3	-6.7, 0.1	0.06	-2.7	-7.2, 1.8	0.24	-2.4	-5.8, 0.9	0.15
Latinx	-0.9	-5.5, 3.6	0.68	-0.7	-6.2, 4.7	0.80	-0.8	-4.2, 2.7	0.67
Multiracial/Other	1.5	-2.9, 5.9	0.51	1.1	-5.7, 7.9	0.74	-1.7	-6.2, 2.9	0.47
**Gender identity**									
Man	—	—		—	—		—	—	
Woman	5.1	2.1, 8.0	0.001	1.5	-2.4, 5.4	0.45	1.8	-1.2, 4.8	0.24
**Education**									
$\le$High School	—	—		—	—		—	—	
Some College	2.2	-0.7, 5.2	0.14	-1.8	-5.7, 2.0	0.35	0.1	-2.8, 3.0	0.94
$\ge$College Degree	-0.3	-4.8, 4.1	0.89	-2.4	-7.4, 2.6	0.35	4.4	1.0, 7.8	0.012
**Employment**									
Other	—	—		—	—		—	—	
Full/part-time	-0.4	-3.3, 2.5	0.79	2.8	-1.1, 6.6	0.15	-2.3	-5.2, 0.6	0.11
**Dependents**									
0	—	—		—	—		—	—	
1	-0.7	-4.3, 2.8	0.68	1.9	-3.0, 6.7	0.45	1.8	-1.5, 5.1	0.29
2 +	-0.8	-4.6, 2.9	0.67	3.5	-1.8, 8.8	0.19	3.4	0.1, 6.7	0.043
No. Obs		190			116			269	

*CI* Confidence Interval

We found that lower financial stress also correlated with older age (37–44 b = -3.5, 95% CI: -5.7, -1.2; 45–57 b = -4.3, 95% CI: -6.6, -2.0; 58–90 b = -7.7, 95% CI: -10.0, -5.1) and Black race (b = -2.9, 95% CI: -5.0, -0.8) in the overall sample. The association of older age, but not Black race, varied by marital status. The difference in financial stress between the 18–36 age group and the 58–90 age group was much larger among married/living with a partner participants (b = -9.7, 95% CI: -13.8, -5.7) than among never married participants (b = -7.3, 95% CI: -12.1, -2.4).

A positive correlation was found between women and financial stress in the overall sample, indicating that women (b = 2.9, 95% CI: 1.1, 4.7) tended to have higher financial stress than men. This association was larger among never married participants (b = 5.1, 95% CI: 2.1, 8.0). Higher education was associated with greater financial stress among participants who were married/living with a partner (b = 4.4, 95% CI: 1.0, 7.8). Lastly, having 2 or more dependents (b = 2.3, 95% CI: 0.1, 4.4) correlated with higher financial stress in the overall sample. This association was greater among participants who were married/living with a partner (b = 3.4, 95% CI: 0.1, 6.7).

## Discussion

Understanding the impact of marriage and social status on financial stress is important for promoting the well-being of FIIs. Analyzing data from the 2023 Survey of Racism and Public Health, we found statistically significant individual and interaction effects of marital and social status on financial stress. Specifically, being married/living with a partner or having higher perceived social status was protective against financial stress. However, the interaction effect suggested that the effect of social status was more protective among those who were divorced/separated/widowed compared to those who were never married and married/living with a partner. Findings suggest that bolstering family cohesion and promoting subjective social status may be important intervention opportunities to alleviate financial stress in this population.

The stronger protective impact of subjective social status on financial stress for divorced/separated/widowed individuals, compared to married or single individuals, may be attributed to various factors. Divorce or loss of a partner often leads to a loss of dual-income households, asset division, and potential legal expenses, creating significant financial burdens (Kelley et al., 2018). However, individuals who may have had higher social status before the marriage may experience significantly less financial stress due to their access to social resources, such as financial resources from an extended social network, emotional support outside of a marriage to overcome struggle, or broader community support.

The trends observed in financial stress for individuals of older ages, as well as respondents who identified as a woman, are consistent with previous literature (Harner et al., 2017; Holden et al., 2021; Sered & Norton-Hawk, 2019). Holden et al. (2021) found that older age was associated with lower financial stress, which aligns with our observations. The higher levels of financial stress that were observed among respondents who identified as a woman, were similarly observed in other empirical investigations (Harner et al., 2017; Sered & Norton-Hawk, 2019). For instance, a longitudinal qualitative study of 37 formerly incarcerated women in Massachusetts found that, over the course of a 10 year follow-up period, none of the women had been steadily employed. This was attributed to substantial health, legal, gendered, and economic barriers to employment (Sered & Norton-Hawk, 2019). Another qualitative study by Harner et al. (2017) reported that common financial stressors among incarcerated women included the inability to afford healthcare, low-wage jobs, and dependence on others. The collective findings from these studies and the present study characterize currently and formerly incarcerated women as a population needing additional attention and support. To further elucidate this gendered experience among FIIs, future studies could examine how this may be linked to the exclusion of individuals with felony convictions from social welfare benefits, as these exclusionary policies are known to have a disproportionate impact on single mothers (Morgan et al., 2023).

Insights from this study highlight the importance of improving health outcomes among FIIs. Chronic financial stress often coincides with prevalent chronic diseases such as asthma, obesity, and hypertension among FIIs (Houle, 2014; Howell et al., 2016; Massoglia, 2008; Visher et al., 2004; Wang et al., 2009). Managing chronic health conditions is expensive (National Center for Chronic Disease Prevention and Health Promotion, 2023), which in turn leads to the majority of FIIs failing to adequately take care of their health needs as they often lack health insurance after release (Massoglia & Pridemore, 2015; Massoglia & Remster, 2019; Visher et al., 2004). In the sample of the current study, 27% (*n* = 127) FIIs had one chronic disease, and 54% (*n* = 317) had two or more chronic diseases. Our study also suggests other stressors among this population such as low perceived social ranking. Studies have shown that financial stress and decreased social standing correlate with unhealthy behaviors, including increased rates of smoking and fast-food consumption among FIIs (Porter, 2014). Although outside of the scope of this study, these unhealthy behaviors, coupled with cumulative stressors before, during, and after incarceration demand immediate attention from health scientists and professionals to prevent high premature mortality rates among this population.

Our findings have several implications for intervention efforts to support the reintegration of FIIs into society in a manner that reduces financial stress and unnecessary burden on marital relationships. For instance, understanding that financial stress levels may vary by marital status, providing tailored couseling or financial literacy programs that are inclusive of spouses/partners of FIIs may improve the quality of social and marital relationships (Doleac, 2018; McKay et al., 2016). Outside of the marital relationship, efforts can also be made to reduce the societal burdens that lead to financial stress. For example, the Case Close Project in New York, supported by The Legal Aid Society, provides legal representation, community education, and advocacy and has successfully sealed hundreds of criminal records since 2017 (The Legal Aid Society, n.d.). Funding and replicating these types of programs across the U.S., has the potential to increase FIIs eligibility for employment opportunities and enable them to apply for federal programs such as the Supplemental Nutrition Assistance Program or Housing Choice Voucher Program (Mele & Miller, 2005).

Additionally, given the challenges associated with securing employment and the resulting financial strain, providing opportunities for FIIs to volunteer their skills in exchange for monetary vouchers to pay off their legal debt can alleviate their compounding criminal justice-related fees (Pager et al., 2022). These include late payments, local and state financial obligations, and public defender costs (Bannon et al., 2010), which serve to increase financial strain and marital stress. Importantly, evidence demonstrates that FIIs who are given the opportunity to help others (e.g., mutual help groups) have higher self-esteem, better psychological well-being, and report greater satisfaction with life (Lebel, 2007). Ultimately, these efforts may buffer the effects of financial strain after incarceration, reduce recidivism, and improve how FIIs perceive themselves in society.

### Limitations and strengths

The findings of this study should be interpreted within the context of several limitations. First, we did not stratify by the length of time spent incarcerated, though this may impact levels of financial and marital strain and subjective social status. Second, we could not differentiate between married individuals before, during, or after their incarceration, which could have been a potential confounder. Third, our sample consisted of FIIs living in Northeastern or Southeastern states and was recruited as a nonprobability convenience sample, thus limiting the generalizability of this study. This self-reported survey was also subject to recall bias. This study should be interpreted as an exploratory analysis, warranting the need for future research with generalizable study designs. Fourth, indicating incarceration history on a survey may be a sensitive topic. This may have led to some FIIs choosing “no” to respond to the survey, potentially excluding them from the analytic sample. Fifth, the cross-sectional associations observed in this study do not imply causal relationships between marriage, social status, and financial stress. Lastly, we did not conduct a longitudinal analysis of financial stress, thus preventing us from exploring trends over time.

Despite its limitations, the present study has several strengths and makes innovative contributions to the literature. We analyzed a large sample (*n* = 588) of FIIs living in Health and Human Services Regions 1, 2, or 3, a densely populated area that accounts for approximately 20% of the US population. In addition, we utilized a multidimensional measure of financial stress (Heo et al., 2020), which has not been widely examined among FIIs. This measure encompasses five constructs: psychological distress, financial anxiety, job insecurity, life satisfaction, and financial well-being (Heo et al., 2020). Furthermore, the present study extends previous research by focusing on the financial strain of the FII, rather than that of the romantic partners and family members (e.g., children) of FIIs (Arditti et al., 2003; Comfort, 2008; Davis, 1992; deVuono-powell et al., 2015; Geller et al., 2011; Grinstead et al., 2001; Johnson, 2009). We also assessed the combined associations of marriage and social status as potential mechanisms producing disparities in financial strain among FIIs. Previous studies have primarily focused on the effect of incarceration on marriage quality or dissolution, with limited follow-up studies on married FIIs during reintegration (Apel et al., 2010; Braman, 2004; Geller, 2013; Lopoo & Western, 2005; Massoglia et al., 2011; Western, 2006; Western et al., 2004; Widdowson et al., 2020). By examining differences in financial strain across marital and social statuses during reintegration, vulnerable subgroups within the formerly incarcerated population can be identified and targeted for intervention. Lastly, we leveraged a novel dataset, the 2023 Survey of Racism and Public Health.

## Conclusion

In conclusion, we provide novel insights into the interrelationship between marriage, social status, and financial stress among FIIs. We demonstrated that perceived social status and social support received through marriage or partnership were protective against financial strain, with varying effects of social status across marital status. Our research also indicates that formerly incarcerated women experience higher financial stress than men, highlighting the need for additional research and targeted programming to support this subgroup. Findings indicate the need for policies and programs which may target the family unit, and not only the individual, to help alleviate the financial burden of FIIs. Finally, programs that offer legal aid to assist in expungement or sealing of criminal records or those offering opportunities for community volunteer work in exchange for vouchers specific to legal debt among FIIs could serve to reduce financial stress and improve social standing.

## Data Availability

The dataset supporting the conclusions of this article can be obtained by emailing a request to the Center for Anti-racism, Social Justice, & Public Health (gph.casjph@nyu.edu).
